# Agricultural Shows, Judges and the Veterinarian*Read before the Pennsylvania State Veterinary Medical Association.

**Published:** 1893-03

**Authors:** R. S. Huidekoper

**Affiliations:** Veterinarian


THE JOURNAL
OF
COMPARATIVE MEDICINE AND
VETERINARY ARCHIVES.

VOL. XIV. MARCH, 1893. NO. 3.

### AGRICULTURAL SHOWS, JUDGES AND THE VETERINARIAN.*

BY R. S. HUIDEKOPER, M.D., Veterinarian.

In the earliest days of which we have written records of the doings of man in connection with the domestic animals, we find the greatest agricultural and medical authorities assuming the functions of the Veterinarian, and in many cases the productions of the Greek and Roman writers attained their greatest celebrity from their knowledge of the diseases of the lower creation. In the middle ages the knight, the nobleman and the physician were proud of their attainments in diagnosing and treating the diseases of the horse, the hound and the hawk, which furnished the demands of war and sport for the better classes of the day. We find the earlier veterinarians of reputation, recognized authorities and advisers of Kings and Ministers in the establishment of governmental breeding studs, in the control of cattle commerce, and in the supervising of legislation in regard to epizootic diseases.

In the present century, when veterinary medicine took rapid strides in advancing the education of the multitude in its science and arts, the agricultural societies of the old country have ever given the Veterinarian his proper place, and have relied upon his judgment for the decision of those more delicate technical points for which he is fitted.

In America, where the veterinary profession has been but recently recognized, except in the sporadic cases where some individual has enforced his own recognition through the merits of his own attainments, the Veterinarian has not been given his proper place in the advisory boards of the agricultural community.

* Read before the Pennsylvania State Veterinary Medical Association.

Perhaps, probably, this has been, to a great extent, his own fault. The schools have taught the elements of medicine, the anatomy of the horse, the physiology of the generative organs of the cow, the pathology of the horse and the pathology of the ox as if these were entirely separate lines of study, and they have given diplomas to students who were sent out to look at animals on the hoof and did not know the differential external points of a thoroughbred stallion from a Clydesdale mare, or a Hereford steer from a Jersey cow, and when it came to sheep and hogs did not know that more than one race of each existed.

Counties hold their agricultural fairs, and the local Veterinarian is not heard of in connection with them; States have greater fairs, and still I do not know of one where the Veterinarian has been recognized officially.

The National Horse Show Association of America (limited), holding shows in New York, has employed three Veterinarians, with the title of "Veterinary Inspectors," at each of their exhibitions, in order to guarantee to the exhibitors proper sanitary supervision, and to have on hand a veterinary attendant in cases of emergency; and these Veterinary Inspectors are required to be within call of the judges in the ring, in case the judges desire professional opinion in regard to any of the animals in the competition.

The Philadelphia Horse Show, organized in 1892, also employed Veterinarians.

The United States Horse and Cattle Show Society (New York), at its Spring show, 1892, employed three Veterinary Inspectors, who examined all horses before they came before the judges, and the regulations required that none but sound horses should be considered. Still, even at this show, which attempted to give the Veterinarian more recognition than he had been accorded before, want of system, too short a time and previous habit did not allow of as rigid an inspection and regard for the veterinary certificates as might be desired. It was, however, a great step in the right direction.

The veterinary profession should be closely allied to all agricultural shows for their mutual interest.

The Veterinarian derives from his connection with agricultural shows a public reputation, a recognition of his position as an expert, an experience which is an education, directly the fee which he receives for his services, and indirectly the practice which it brings him in the future.

The agricultural society and the public derive from the employment of an expert veterinarian a judgment of the animals exhibited which can only be had from one who has made the subject his life's work; they are able to be perfectly satisfied that their awards have not gone to undeserving animals, and posterity is benefited by the elimination of animals from the "blue book" of breeding which would be detrimental to the future stock of the country.

The position of the Veterinarian in agricultural shows in America is still, however, a tentative point, and in order to establish it the duties of the judges should be first well defined.

The object of an exhibition of animals is to bring together animals of certain classes which are fitted to fulfill the purpose for which they are intended, and to decide upon the relative merits of such animals as conform to the requirements of their class. An animal which may be perfect in one class may be worthless in another. A thoroughbred mare of golden pedigree may be a grand stake winner, and if she has a neoplasm in the mammary glands may be unfit for a broodmare; a Durham bull may be the type of a shorthorn winner, and if he is impotent may not be eligible for a breeding class; a setter dog may be a picture of perfection, and if he is gun-shy cannot compete in a field trial; and the most beautiful cock ever seen, if a coward, is not fit for the pit.

The rules and regulations of the prize list should define distinctly what is demanded, and it is, first, the duty of the exhibitor to determine if his animal is eligible for competition; second, to enter it properly. It is then the duty of the judges to decide, first, if the animal is eligible, and, secondly, upon its comparative eligibility in the class in which it is shown.

For this we must divide our animals into their respective classes, according to their species, races and the service for which they are intended. Following the ordinary classification of veterinary text-books on anatomy, we have: The horse, horned cattle, the sheep, the goat, the hog, the dog, poultry.

In each of these classes we have the various races of the species which offer qualities of conformation and functional abilities as diverse in some cases as the species are, one from another.

In the horse there is the heavy draft, intended to pull the greatest loads of a city traffic, and lighter draft, which must pull the omnibus at a trot. The thoroughbred which wins at four furlongs may have the same breeding, but is as far removed from the horse of the same breeding which can run a four-mile heat race as is the infant's pet, a Shetland pony, from a perfection of a hackney mare, and yet the two latter are relations.

The Hereford steer dressing 1,400 lbs. of beef or a brawny Polled Angus bull, and a Dutch cow which gives twelve gallons of milk a day and a Jersey cow which gives twenty-eight pounds of butter a week, are all horned cattle, and still they require as different judgment as is needed for the beautiful mutton qualities of a flock of Southdowns and the silky fleece of a lot of Rambouillet Merinos.

Certain general principles govern the methods of adjudication of the merits of all animals:

1st. *Race type*, showing the character of the class under consideration.

2d. *Conformation*, regulated by the demands of the type exhibited.

3d. *Individuality*, by which the special merits of each animal examined are compared with those of its competitors.

The determination of the race type demands the educated experience of the expert, who must be cognizant of the origin and history of the race, of the interbreeding and modifications which have been produced by the fashion of modern times, and of the effects of locality which are capable of making important changes in the character of even primative types.

Race type requires still closer scrutiny; here the modifications produced by civilization, and the artificial methods exercised by man in adapting animals to his uses, makes the lines between distinct families still more difficult to determine. The very adaption of lines of breeding to specific purposes to suit the taste of the breeder, generates in the expert a prejudice in favor of that special *genre* to which he has been devoting himself, and still it renders the honest expert more acute to recognize the differential points of animals under consideration, and allows him, when governed by rules for judging, to lay aside personal feeling and find in the very faults, from individual point of view, winning qualities in classes not favored by himself.

JUDGING HORSES.

The classification of horses is based upon their destination as breeders or as motors, and yet while in some classes the points demanded are absolutely different, in others they are so closely allied that the one cannot be thought of without the other. A short resumé of the points to be considered will make the references to the characteristics of the various races more easily understood.

*The Head.* The head by its shape, profile of face, breadth of forehead, angle of occipital bone and development of jaw, is characteristic of race in a breeding class, and evidence of intelligence in a utility class.

*The Neck.* The shape and size of the neck is a sign of quality, and its valuation is absolutely dependent upon the utilization of the animal. While the horse used for stud purposes is handsome, with a thick, curved and solid neck, the mare or gelding intended for a lady's victoria must have one as thin as that of the swan, and all judgment of this part of the body must be secondary to the other more essential parts.

*The Shoulders.* The shoulders, comprising the shape of the withers, the development of attachment of the muscles from the neck and the body, the angle of the scupula to the horizontal axis of the body and the development of bone and muscles of the shoulder blade itself, are evidence of utility rather than of race, and yet certain *families* demand the characteristic of shoulder because their type is based upon their utility, as is exemplified in the Hackney (Mr. Cassatt's mare, Plaisanterie, or Mr. Lawrence's horse, Fashion), where the action could not be obtained without the proper anatomical conformation.

*The Arm.* The arm must conform secondarily to the shoulder, and must be muscled according to its surroundings.

*The Forearm.* This organ is not usually given sufficient consideration. Many an apparently bad shank, fetlock and foot is blamed when the fault lies in the defective aplomb of the forearm, which slants forward or backward when its center of gravity should be almost perpendicular. The development of the muscles varies according to race, which must always be taken into consideration.

*The Knee.* In judging, the knee is included in the other structures below, known as the anatomical foot. It should be broad and flat, the prominences of each individual bone well developed and be free from blemishes.

*The Shin.* The metacarpal region can be good in such varied forms that the anatomist becomes desperate when he studies clinical results. The proper shank has a clean, wide bone with the three rows of posterior tendons well separated, so that the leg at this point is broad from in front to behind, and forms a flat plate from side to side, and it should form a vertical pillar between knee and fetlock. And, yet, race colts bring thousands of dollars with shanks as bent as those of a broken-down post horse. Draught horses with splints, which make the bone as knotty as an Irish shillalah, do the best of work, and the type of a perfect bone "bucks" at the first training.

*The Fetlock and Pastern.* These parts are somewhat subject to the general character of the horse, but should always be strong, evenly set, well developed at the bony points of tendinous attachments and free from lesion.

*The Surgical Foot* (the hoof) should be

Shaped to suit the animal it carries.
Sound in texture.
Support on ground level.
Symmetrical.
Satisfactory to examiner.

It is not in place here to go into the anatomy of the foot, and many shapes of feet are admirable working organs, but the above classification must be followed in judging. Great flat feet are characteristic of the Clydesdale, Shire, Dutch Draft and Percheron, and while they may still be good feet with considerable variation in size and shape, a small, high-heeled hoof arouses suspicion of malformation, and calls for a close examination of the bony parts subjacent.

The texture of the hoof must be good, but allowance can be made for the general condition of the animal and for the evidence of accidental injury and want of care on the part of the owner.

The evenness of support on the ground is a matter of expert judgment only. An abominably crooked foot in a mule-hoofed thoroughbred may not be considered an unsoundness or a hereditary disease, while a much less deformed foot in a Shire would condemn the animal.

There should be a well-established symmetry in the size and shape of the feet on a horse. The fore and hind feet should be relatively well proportioned. The pair of the anterior and posterior biped should be symmetrical. One narrow, small foot may allow the animal to travel sound, may have been acquired accidentally, or may have "been foaled so," but in any case it promises a continuance in the progeny of a breeding animal.

Finally, a considerable amount of latitude in regard to the feet must be given to the expert examiner, whose experience has taught him what is useful and what is not.

*The Body.* No matter what the class of horse may be, the shape of a good body demands depth of the chest, free, open arching of the ribs, ribs well back to protect the abdomen and a regular movement indicating a normal condition of the organs contained under them.

The "tucking" of disease, or the reduction of the posterior part of the barrel produced by training must be differentiated from the congenital shad belly of many "crocks" which would otherwise pass for fair horses.

*The Croup.* The shape of the croup, no matter what is the condition of nutrition of the animal, is based entirely upon the conformation of the bony pelvis and is in all pure breeds a distinction of race. The long, horizontal croup is therefore essential in the Arab, thoroughbred and their crosses, while the Irish horse, with his long, angular croup, and the Shire with his short, angular croup, transmit their characteristics to the hackney and form a type. The set of the tail in pure races coincides with the form of croup. The thigh and leg in well conformed horses also correspond to the form of croup, but when it comes to individuals the most varied results are sometimes obtained by animals with closed coxo-femoral angles and open femoro-tibial, and vice-versa.

*The Hock.* The hock admits of only two decisions for the lay judge, good or bad; it is a good hock and in conformity with the general development of the animal, or it is a bad hock, because it is badly made, or is not in conformity with the other structures of the animal. No relative estimation of the value of a hock can be made by any one but an anatomist and experienced clinician.

The hind foot is estimated by the same general rules as are the anterior homologues.

After the conformation of the horse has been examined sufficiently to determine the race type of the animal and the grade of perfection which the individual attains in it, there comes the estimation of relative merit, which is based upon quality and individuality.

*Quality.* By quality we understand that *ensemble* of purity of type, condition of organs and functions which permits of the anatomical structure being used to advantage, and a development of the nervous system, brain power and healthy periferal sensory and motor nerves which permits the horse to control and exert all of its energies.

The valuation of quality is, to a certain degree, a matter of opinion based upon experience, and it demands considerable experience to determine it in all cases. The novice of good taste the artist who has never seen a horse, can appreciate an animal of perfect quality, whose every pose at station and every symmetrical movement in action denotes grace, strength and intelligence. But in the examination of dealers' horses just off of the cars, brood mares heavy in foal, animals rough and uncared for, just up from pasture, or animals deteriorated by illness, it requires not only a careful appreciation of the conformation, but also of the functional activity of which the individual part is capable and a rigid comparison of the harmony which exists between these.

Individuality furnishes the final basis for appreciation of the comparative perfection of the development of form and quality which is exhibited by the animal.

*Manners.* Another element which plays an important part in the estimation of animals, but which has a variable value according to the use for which animal is intended, is manners. By manners we mean the evidence of education which the animal has received, which fits it for the use for which it is intended. A stallion for breeding purposes need only be so bridle-broken and controllable in the hands of the groom that he can be exercised and led to service with comfort and safety to himself, the mare and the attendant; a saddle horse must be willing to be mounted quietly, be bridle-wise and always ready to take the gait desired by a capable rider; the harness horse must be kind and steady at the various unexpected sights and sounds of the public highways; the draft horse must find pleasure in "collaring" heavy loads and working long hours; the unbroken yearling, or the broncho just roped on the plains, has manners enough, if it is not vicious.

As horse shows are intended to educate the people in the knowledge of the good qualities of the horse, and to stimulate the breeding and development of good animals by the awarding of prizes to those which best conform to a proper standard of merit, it is essential that the judging and awarding of prizes should be done with intelligence and fairness by those who are thoroughly competent.

In the many classes into which horses are divided, according to the use for which they are intended, it is necessary that judges should be selected who have both a general knowledge of all races of horses, and a special knowledge of the class for which they are selected.

The judges estimate the type, conformation, quality and manners of the animals, receiving them with certificates from the veterinary inspectors, who have only to do with the soundness of the animals.

Notwithstanding American custom of bringing in the Veterinary judge last to support or conflict with the opinion of the other judges upon some specific point, his place should be an initiatory one, as it is in the old country. All horses before entering the show ring should have been examined, for the satisfaction of the owner, by some reputable veterinarian. This, in ordinary cases, protects him from disappointment, and prevents the entry of horses which are not eligible for competition from unsoundness. The veterinary inspectors of the show should examine the animals in separate paddocks, where the inquisitive and meddlesome outsider is not admitted, and they should furnish a written certificate for the judges, which should accompany each animal, and which should be final. The veterinary examination is based, as it would be in private practice, upon the destination of the animal. A breeding animal may be broken down or have serious blemishes which would unfit it for work, but it must be free from hereditary unsoundness. Custom has established the hereditary diseases to be the following: roaring, whistling, ringbone, spavin, navicular disease, cataract.

In the utility classes, while, of course, an absolutely clean sound horse, should always take precedence over one which has, blemishes, yet animals with blemishes which do not, or are not liable to produce lameness or interfere with their usefulness, can be passed as practically sound or serviceably unsound, and be considered for competition.

When the horse has passed the veterinary examination and received its certificate of soundness, it goes before the regular judges, who should accept the certificate, and have only to do with conformation, quality and manners, and such specific conditions as are called for in the various classes by the prize list.

The judges of horse shows in America have, at various times, been selected for very arbitrary reasons—presumably they should always be selected for their experience and competence—but unfortunately there is often a rich stockholder of a county fair who is flattered by being considered a horseman, and who repays the compliment by settling the society's deficits; or managers of shows take greater interest in their personal friends than they do in the public good; or a social element enters, and a gentleman recognized for his integrity, whose knowledge of horses is based solely upon the type of his own horses and those of his friends, who belong to the same riding or driving club, is appointed in preference to the man whose experience and ability has been acquired by a life of practical horsemanship or of veterinary practice, which has trained him to recognize the qualities of all classes of horses, whether they are ideals of his own type or not. The large American shows have, however, shown a decided improvement in the last few years in the selection of judges, and the veterinary profession has been recognized by the selection of Professor McEachran, of Montreal, and Professor Smith, of Toronto, as judges in a number of classes.

With the increasing number of classes of animals for which prizes are given, and the growing volume of good animals entered for exhibition, together with the host of "crocks" who find entrance to shows through the vanity of their rich owners, sufficient time cannot always be had for complete inspection, and it is often bewildering to pass many animals rapidly in review and attempt to remember their various qualities. Even with a small number of animals in competition, no man can accurately remember the details of conformation and quality upon which he should base his comparison of relative merit, and the most conscientious and honest of experts, when he attempts to carry the *ensemble* of an animal in his mind, must be more or less governed by his own prejudice or predilection in favor of some type or resemblance to some animal which has been a paragon in his own stable, or past experience.

For accurate, unbiased judging, especially when a number of animals are in the competition, a standard should be established and a scale of points should be used by which each detail, and the aggregation of details, may be estimated, and thereby a definite result reached. This method of judging renders the work much easier and more rapid for the professional judge, and both educates the novice or amateur and leads him to learn the points he should look for.

The following is my own scale of points, which I recognize to be incomplete, but which I am glad to bring before the public, inviting its criticism and asking for suggestions in order to perfect it and make it more useful. It will be seen that in 100 points, 60 points are allowed for conformation and 40 points for quality and manners, which allows the judge ample latitude for generosity to his own expert appreciation and personal opinion.

By grading the judgment on a scale of 5 for perfection to 1

or o for very bad, and multiplying the value, it will be seen that the perfect horse would be marked with 500 points, and others attain their relative place.

SCALE OF POINTS IN 100.

| | | Value in 100. | Revised Value. | Degree of Perfection. | Definite Value. | Remarks |
|---|---|---|---|---|---|---|
| | | | | 1–5 | Total 500 | |
| Head | Conformation 60 | 4 | | | | |
| Neck | | 2 | | | | |
| Shoulder | | 6 | | | | |
| Arm and Forearm | | 2 | | | | |
| Anatomical foot<br>Knee to pastern, anterior. Below hock, posterior. | | 6 | | | | |
| Surgical foot<br>Hoofs, anterior and posterior. | | 10 | | | | |
| Body | | 10 | | | | |
| Croup | | 6 | | | | |
| Thigh and Lower Thigh | | 4 | | | | |
| Hock | | 10 | | | | |
| Quality<br>Quality proper.<br>Action fore.<br>Action hind.<br>Pedigree. | | 25 | | | | |
| Manners | | 15 | | | | |

MODIFICATION OF SCALE.

It will be readily understood that the various parts have not the same value in all classes of animals, and the blank second column and column of remarks are inserted for adjusting the table to the classes under consideration.

*Head* (*value 4*). In breeding classes the head is important as an evidence of race type. It is essentially so in all of the primitive races, as the Oriental horse (Arab, thoroughbred, Barb) ; the Irish horse (Shetland, Breton); the Shire; the Dutch horse (Clydesdale); the Danish horse (Normans, Luxembourg). In families of mixed race, like the hackney, the standard-bred trotter, etc., it is of considerable value as indicating the predominence of ancestry in the formation of the *family*. In common horses its value is included in quality according to the amount of intelligence shown.

*Neck* (*value 2*). The neck is given a value of two points only, as the estimation of its conformation must always be subservient to that of the head and withers, which it joins.

*Shoulders* (*value 6*). The shoulder, including the withers, in many cases, demands a greater value than the six allotted to it.

The speedy race horse or trotter or the high-stepping hackney demand more value for this part. The saddle horse requires more consideration for the withers than the ordinary harness horse.

*Arm and Forearm* (*value 2*). These are given but little value, as they are subservient to the shoulder and their aplomb is dependent to a certain degree upon the foot below.

*Anatomical Foot* (*value 6*). The value of the anatomical foot is based upon the conformation, aplomb, relative angles and soundness of the knee, cannon bone, splint bones, tendons behind the shin and of the fetlock and pastern.

*Surgical Foot* (*value 10*). The hoof must not only be good in itself, but must be adapted to the animal it carries, and ten points are none too many for any animal which is to perform work or be used for a breeder.

*Boay* (*value 10*). The barrel, like the feet, in any horse must be good, as any defect interferes with the function of respiration or diminishes the space for food.

*Croup* (*value 6*). This value is none too great in pure races, where it is an evidence of race type, and is given this value in speedy breeds, although in the latter we may find fast horses with all forms of croup. The set of the tail is a race characteristic in some animals (Shetland, Hackney, Shire), and is of value in all better bred horses and animals of luxury, where it is an ornament.

*Thigh and Lower Thigh* (*value 4*). These, like the arm and forearm, are dependent, to a great degree, upon their relations to the structures above and below, but have much individual value of their own.

*Hock* (*value 10*). While this part deserves the full valuation of points given to it, it is the one which will most rarely be accorded its full number, and care should be taken not to award the decimal to any but the most perfect of bold, strong joints, which should never go wrong.

*Quality* (*value 25*). Quality may be subdivided into : Quality proper, action of fore legs, action of hind legs, pedigree ; and, according to the class of animal examined, a certain part of the 25 points may be accorded to each. So an animal in a high-stepping class may have 8 for action fore, 8 for action hind and 9 for quality proper, while a thoroughbred stud horse can have 10 for quality and 15 for pedigree. Quality must be most variously interpreted. The peacock head and tail, rangy action and pride of pose of Mambrino King, Bonfire or Bersecker shows quality very differently from the resolute, firm, steady movement which indicates it in a draft horse or mule, but it is of as much value in the latter as in the former.

*Manners* (*value 15*). Manners in trotting classes and in work classes may include the record of performances where the conditions of the competition call for them. The manners of a stud horse are to be easily handled. The manners of a car horse are to keep his neck in the collar and drudge willingly. Young animals are not expected to have any. Some animals, therefore, should have half or more of the 15 points taken off of manners and added to conformation. On the other hand, the four-in-hand team, the pair to a lady's spider, the park hack, or the hunter must be given full credit for them.

